# Supplementary material for: The PavMYB.C2-UFGT module contributes to fruit coloration via modulating anthocyanin biosynthesis in sweet cherry
Source: PLoS Genet. 2025 Jun 17;21(6):e1011761. doi: 10.1371/journal.pgen.1011761 (PMC12185008; doi:10.1371/journal.pgen.1011761)
Supplement: S1 Fig — (A) Relative abundances of various anthocyanins in the fruits of two cultivars at different ripening stages. Different classes of metabolites are represented by distinct colors. (B) Colour of the four main anthocyanins Cyanidin-3-O-rutinoside (Cy3R), Cyanidin-3-O-glucoside (Cy3G), Peonidin-3-O-rutinoside (Pn3R), and Pelargonidin-3-O-rutinoside (Pg3R) at the same concentration (100ug/ml). (PDF) [file pgen.1011761.s001.pdf]

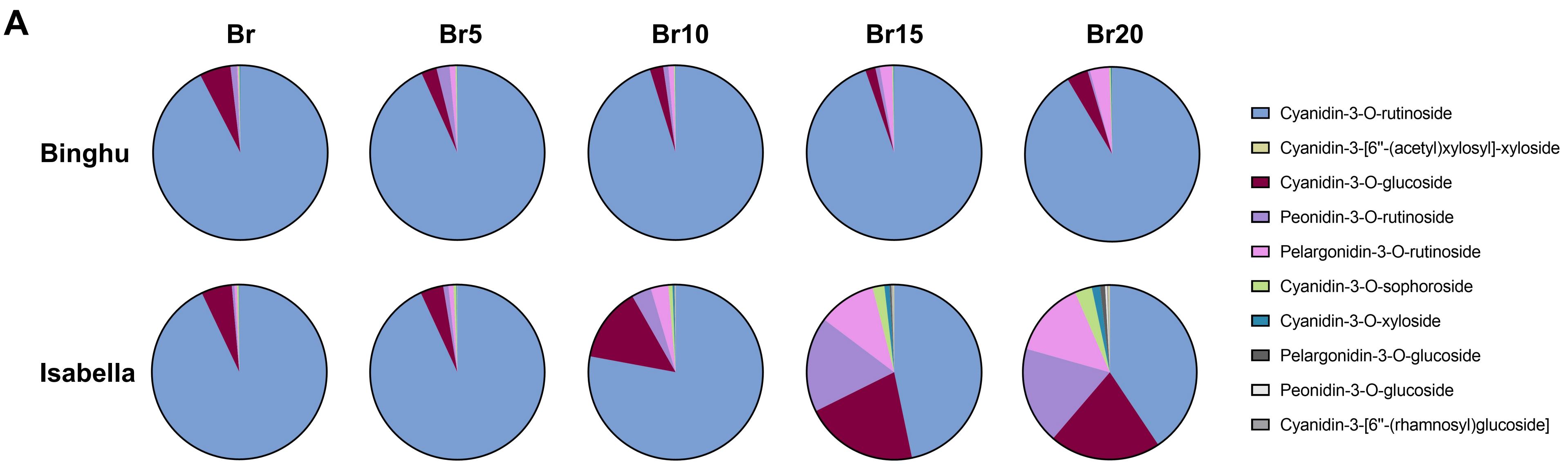

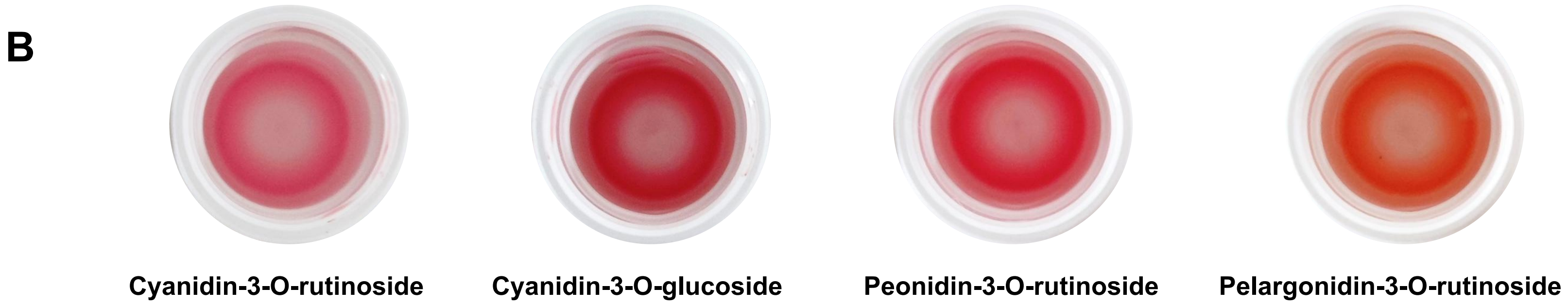

S1 Fig. Comparison of different classes of anthocyanoside substances

(A) Relative abundances of various anthocyanins in the fruits of two cultivars at different ripening stages. Different classes of metabolites are represented by distinct colors.

(B) Colour of the four main anthocyanins Cyanidin-3-O-rutinoside (Cy3R), Cyanidin-3-O-glucoside (Cy3G), Peonidin-3-O-rutinoside (Pn3R), and Pelargonidin-3-O-rutinoside (Pg3R) at the same concentration (100ug/ml).
