# Supplementary material for: The PavMYB.C2-UFGT module contributes to fruit coloration via modulating anthocyanin biosynthesis in sweet cherry
Source: PLoS Genet. 2025 Jun 17;21(6):e1011761. doi: 10.1371/journal.pgen.1011761 (PMC12185008; doi:10.1371/journal.pgen.1011761)
Supplement: S2 Fig — (A-C) Volcano diagrams of DEGs in ‘Isabella’ and ‘Binghu’ fruits. A, Br10 stage; B, Br15 stage; C, Br20 stage. (D-F) KEGG pathway analysis of DEGs in ‘Isabella’ fruits compared to ‘Binghu’. D, Br10 stage; E, Br15 stage; F, Br20 stage. (PDF) [file pgen.1011761.s002.pdf]

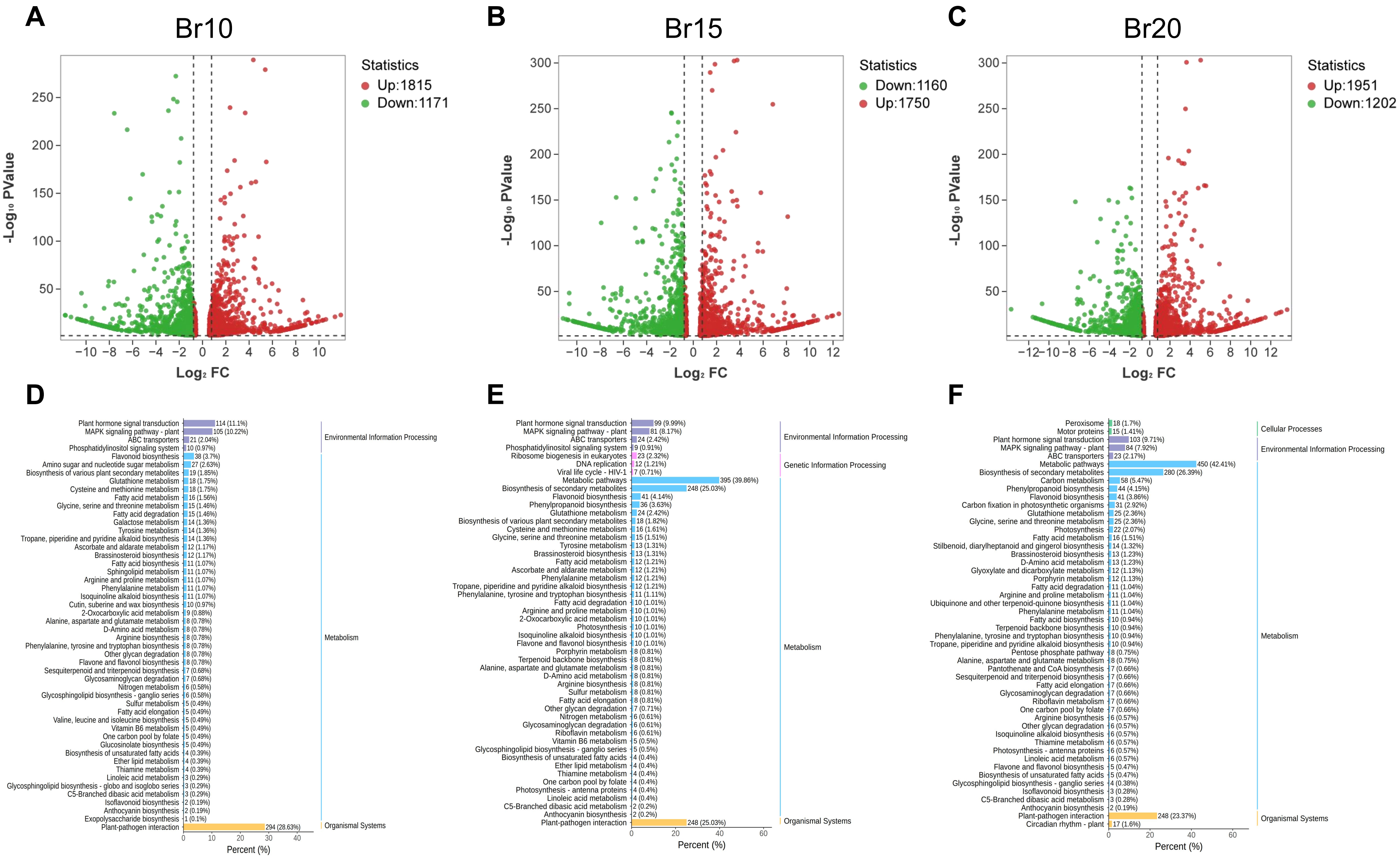

S2 Fig. Analysis of DEGs between two cultivars under ripening stages

(A-C) Volcano diagrams of DEGs in ‘Isabella’ and ‘Binghu’ fruits. A, Br10 stage; B, Br15 stage; C, Br20 stage.

(D-F) KEGG pathway analysis of DEGs in ‘Isabella’ fruits compared to ‘Binghu’. D, Br10 stage; E, Br15 stage; F, Br20 stage.
