# Supplementary material for: The PavMYB.C2-UFGT module contributes to fruit coloration via modulating anthocyanin biosynthesis in sweet cherry
Source: PLoS Genet. 2025 Jun 17;21(6):e1011761. doi: 10.1371/journal.pgen.1011761 (PMC12185008; doi:10.1371/journal.pgen.1011761)
Supplement: S3 Fig — (A) Phylogenetic tree constructed using amino acid sequences of MYB domains. (B) Phylogenetic tree constructed using full-length coding DNA sequence. (PDF) [file pgen.1011761.s003.pdf]

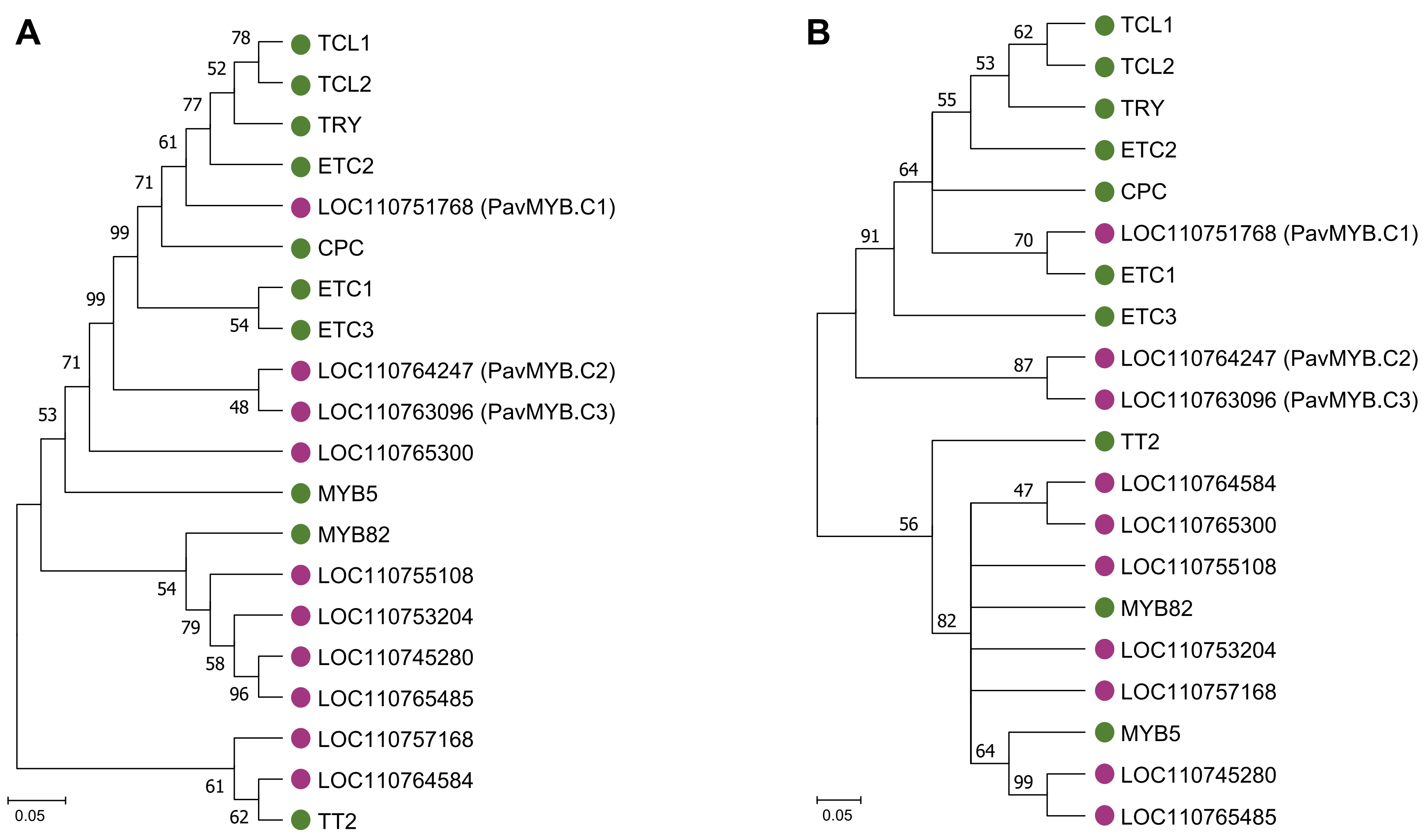

S3 Fig. Phylogenetic analysis of the LOC110764247 (PavMYB.C2) homologs in *Arabidopsis thaliana* and *Prunus avium*.

(A) Phylogenetic tree constructed using amino acid sequences of MYB domains.

(B) Phylogenetic tree constructed using full-length coding DNA sequence.
