# Supplementary material for: The PavMYB.C2-UFGT module contributes to fruit coloration via modulating anthocyanin biosynthesis in sweet cherry
Source: PLoS Genet. 2025 Jun 17;21(6):e1011761. doi: 10.1371/journal.pgen.1011761 (PMC12185008; doi:10.1371/journal.pgen.1011761)
Supplement: S4 Fig — (A) PCA dendrogram of transcriptome data from MYB.C2-OE and control fruits. The oval represents the 95% confidence interval. (B) Volcano diagrams of DEGs in MYB.C2-OE fruits compared to control fruits. (PDF) [file pgen.1011761.s004.pdf]

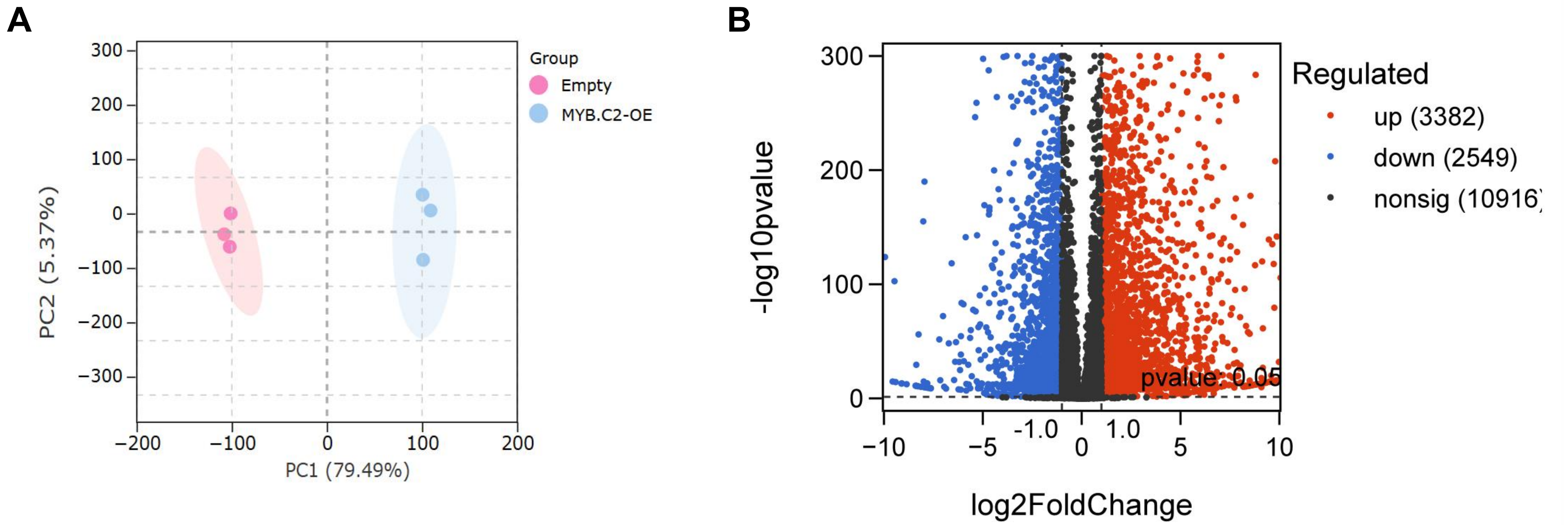

S4 Fig. Transcriptome analysis of *MYB.C2*-OE and control fruits

(A) PCA dendrogram of transcriptome data from *MYB.C2*-OE and control fruits. The oval represents the 95% confidence interval.

(B) Volcano diagrams of DEGs in *MYB.C2*-OE fruits compared to control fruits.
