# Supplementary material for: The PavMYB.C2-UFGT module contributes to fruit coloration via modulating anthocyanin biosynthesis in sweet cherry
Source: PLoS Genet. 2025 Jun 17;21(6):e1011761. doi: 10.1371/journal.pgen.1011761 (PMC12185008; doi:10.1371/journal.pgen.1011761)
Supplement: Attachment — Submitted filename: Response to Reviewers.doc [file pgen.1011761.s014.doc]

**Answers to Editors**

Thank you for submitting your manuscript to PLOS Genetics. After careful consideration, we believe it meets PLOS Genetics's publication criteria, pending revision and re-review. Therefore, we invite you to submit a revised version of the manuscript that addresses the points raised during the review process.

**Answer:** We are grateful to you for your appreciation of the value of our work. To address the reviewers' comments and concerns, we have performed and included additional experimental data to support our claims and ensure the reliability of our findings.

Additional Editor Comments (if provided): All reviewers appreciate your work, and there is strong consensus that the presented study is of broad interest. Please carefully review all the comments. As Reviewer 1 pointed out, please clarify the potential uniqueness of the MYB TF, likely with additional tests. Please submit your raw omics data to any public depository. Please do not hesitate to contact me if you need futher clarifications.

**Answer:** Thank you for your positive feedback and the constructive comments. To further substantiate the potential uniqueness of PavMYB.C2 in regulating anthocyanin synthesis in sweet cherries, we conducted additional analyses and experiments.

First, we performed a comparative analysis of the amino acid sequences of PavMYB.C2, AtCPC, AtTRY, AtETC1, and other CPC-related homologous proteins in *Arabidopsis*. Our analysis revealed two amino acid positions, D34 and M68, within the MYB structural domain that are highly conserved in *Arabidopsis* MYBs, but differ in PavMYB.C2, where D34 is substituted with T34 and M68 with S68 (See Fig A below; Fig. 9F in our revised manuscript). Previous studies have shown that mutations in conserved amino acid residues within MYB domains can lead to significant alterations in their functional properties, underscoring the importance of these conserved sequences (Guehmann et al., 1992, *Nucleic Acids Res* 20: 2279-2286; Yang et al., 2021, *Plant Physiol Biochem* 162: 124-136; Wang et al., 2022, *Nat Plants* 8: 1108-1117).

The [DE]Lx2[RK]x3Lx6Lx3R sequence, located within the MYB domain, is a critical region for the interaction of R3-MYBs with bHLH proteins. Notably, D34 represents the first amino acid of this sequence (Simon et al., 2007, *Dev Biol* 311: 566-578; Matsui et al., 2008, *Plant J* 55: 954-967). Additionally, M68 site has been shown to be essential for the function and intercellular movement of CPC proteins (corresponding residue M78 site in CPC) (Kurata et al., 2005, *Development* 132: 5387-5398). Therefore, we hypothesize that the variations at these two loci may contribute to the functional specificity of PavMYB.C2 in sweet cherry, potentially modulating its role in anthocyanin regulation.

Subsequently, we generated two mutated versions of PavMYB.C2: PavMYB.C2_D34, in which the amino acid T34 was substituted with D34, and PavMYB.C2_M68, in which the amino acid S68 was substituted with M68 (See Fig B below; Fig. 9G in our revised manuscript). These mutated constructs were then introduced into the pGreenII 62SK vector as effector constructs. Both constructs were co-transfected into tobacco leaf protoplasts along with the proUFGT:LUC reporter plasmid, respectively. Our results indicated that co-transfection of the proUFGT:LUC reporter plasmid with the 35S:PavMYB.C2 or PavMYB.C2_D34 plasmid resulted in a significant increase in relative LUC activity compared to the control. However, when the 35S:PavMYB.C2_M68 plasmid was co-transfected, the transcriptional activation capacity was absent (See Fig C below; Fig. 9H in our revised manuscript). These findings suggest that the activation of *UFGT* by PavMYB.C2 is dependent on the S68 site, rather than the T34 site. Based on these data, we propose that the functional specificity of PavMYB.C2 in sweet cherry may be attributed to the variations at the S68 site, which play a critical role in its ability to regulate anthocyanin biosynthesis.

We have now integrated these findings into the results section and have made corresponding modifications to the methods. Additionally, the functional specificity of PavMYB.C2 is further discussed in the discussion section of the manuscript.

The raw omics data reported in this paper have been deposited in the National Genomics Data Center (NGDC), China National Center for Bioinformation under the accession numbers CRA021570 and OMIX009876.

**Regulation of the *UFGT* by PavMYB.C2 is dependent on the S68 site of PavMYB.C2.** (A) Multiple protein sequence alignment of PavMYB.C2 and related MYBs from *Arabidopsis*. The red boxes highlight amino acids that are conserved in *Arabidopsis* MYBs but differ in PavMYB.C2. D: Aspartic acid; T: Threonine; M: Methionine; S: Serine. (B) Diagrams showing the PavMYB.C2 variants. PavMYB.C2_D34, a mutated version of PavMYB.C2 with the amino acid T34 substituted by D34; PavMYB.C2_M68, a mutated version of PavMYB.C2 with the amino acid S68 substituted by M68. (C) Effects of PavMYB.C2, PavMYB.C2_D34, and PavMYB.C2_M68 on the activation of *UFGT*. The empty pGreenII 62SK vector served as the negative control.

**Answers to Reviewer #1**

Based on the pattern of transcriptional regulation anthocyanin biosynthesis is conventionally divided into two stages, early and late stage. Genes belonging to the early stage of the anthocyanin biosynthesis is predominantly controlled positively by R2R3MYB type of transcription factors, while the genes in later stage (including UFGT) in controlled by the MBW complex, comprising R2R3MYB-bHLH-WD40 transcription factor. CARPRICE (CPC) CPC belongs to the R3-MYB subgroup, meaning it has only a single MYB DNA-binding domain. R2R3-MYBs, which typically activate gene expression either single or as part of the MBW complex. Unlike R2R3 MYB, CPC acts as a repressor of certain MYB-regulated pathways, including anthocyanin biosynthesis, trichome, and root hair development. CPC lacks transcriptional activation domain and functions by competing with R2R3-MYBs for binding to bHLH proteins, preventing the formation of the MBW complex needed for activating late anthocyanin biosynthetic genes like UFGT.

From the result provided in the manuscript, it is assumed that PavMYB.C2 belongs to the R3-MYB subgroup. Does PavMYB.C2 contain one repeat or two repeats? This is a unique case where a R3MYB factor can directly bind DNA and positively regulate gene expression, especially UFGT, a gene belonging to the very late stage of anthocyanin biosynthesis. A wealth of knowledge available regarding the transcriptional regulation of anthocyanin in different plants. These are not discussed in the manuscript.

**Answer:** Thank you for your thoughtful comments. PavMYB.C2 is indeed classified as an R3-MYB transcription factor, and it contains a single MYB domain. This highlights the unique function of PavMYB.C2 in the anthocyanin biosynthesis of sweet cherry, which different from the typical regulatory roles observed for R3-MYB proteins in *Arabidopsis*. We have now performed additional analyses and experiments to further demonstrate this species-specific function. Additionally, we have included a more comprehensive review of the transcriptional regulation of anthocyanin biosynthesis across different plant species in the Introduction section.

I would suggest test the transcriptional activity of the PavMYB.C2 on the UFGT promoter where “TGGTAA” site is mutated.

**Answer:** We sincerely thank the reviewer for the insightful and valuable suggestion. In response, we have generated a mutant version of *UFGT* promoter, referred to as *mUFGTpro*, in which the “TGGTTA” binding site was substituted with “AAAAAA”. Then we conducted a dual-luc assay in tobacco protoplasts. Our results showed that co-transfection of the 35S:PavMYB.C2 effector with the mUFGTpro:LUC reporter did not result in a significant change in luciferase (LUC) activity, when compared to the control effector (See Fig below; Fig. 9A in our revised manuscript). This result further suggests that PavMYB.C2 regulates transcriptional expression of the *UFGT* through binding to the "TGGTTA" site.

**Effect of PavMYB.C2 on transcriptional regulation of the *UFGT* promoter following mutation of MBS3 (TGGTTA mutated to AAAAA).**

Does As mentioned earlier, DNA binding in MYB transcription factors primarily depends on the R2 and R3 MYB repeats, which together recognize and bind to specific promoter sequences. A Single-repeat MYBs (R3-MYBs). Such as CPC, TRY, ETC1/2 only have the R3 domain, which alone is not sufficient for stable DNA binding. Additional experiments are required to test the proposed model. Despite species-specific variations, the core regulatory network involving MYB, bHLH, and WD40 are highly conserved across plants. I would suggest that the author should also test whether CPC/TRY/ETC can activate the UFGT promoter in a similar fashion. Also in vitro binding capacity should be tested. If the data presented in this manuscript are accurate, they have the potential to significantly enhance our understanding of the mechanisms by which MYB transcription factors regulate gene expression. These findings may provide new insights that could refine or even reshape existing models of MYB-mediated transcriptional regulation.

**Answer:** We sincerely appreciate the reviewer for bringing up this important point. To investigate whether *Arabidopsis* CPC, TRY, and ETC proteins can activate the *UFGT* of sweet cherry. We introduced *AtCPC*, *AtETC1* and *AtTRY* into the pGreenII 62SK vector as effector constructs (See Fig A below). These constructs were co-transfected into tobacco leaf protoplasts along with the proUFGT:LUC reporter plasmid, respectively. However, we did not observe any significant increase in luciferase (LUC) activity compared to the control (See Fig B below), suggesting that *Arabidopsis* CPC-related proteins do not regulate *UFGT* expression in sweet cherry.

To further examine the DNA-binding capacity of these CPC-related proteins to the *UFGT* promoter, we also performed EMSAs experiments. A biotin-labeled DNA probe containing the TGGTTA site from the *UFGT* promoter was incubated with purified GST-AtCPC, GST-ETC1, and GST-AtTRY proteins, respectively. The results indicated the presence of free-probe, but no electrophoretic mobility shift was observed, suggesting that these CPC-related proteins do not bind to the *UFGT* promoter (See Fig C-E below). Taken together, there results implies that CPC homologs may not perform exactly similar functions across different plant species.

***Arabidopsis* CPC-related proteins do not regulate *UFGT* in sweet cherry.** (A) Diagram of the effectors and reporter constructs used in the dual-luciferase assay. (B) The CPC/TRY/ETC proteins do not activate the transcription of *UFGT*. The relative LUC/REN ratio reflects transcriptional activation activity. The empty pGreenII 62SK vector served as the negative control. (C-E) EMSAs showing no binding of CPC/TRY/ETC to the *UFGT* promoter. C, added recombinant purified GST-AtCPC; D, added recombinant purified GST-AtETC1; E, added recombinant purified GST-AtTRY.

To further substantiate the potential uniqueness of PavMYB.C2 in regulating anthocyanin synthesis in sweet cherries, we conducted additional analyses and experiments.

First, we performed a comparative analysis of the amino acid sequences of PavMYB.C2, AtCPC, AtTRY, AtETC1, and other CPC-related homologous proteins in *Arabidopsis*. Our analysis revealed two amino acid positions, D34 and M68, within the MYB structural domain that are highly conserved in *Arabidopsis* MYBs, but differ in PavMYB.C2, where D34 is substituted with T34 and M68 with S68 (See Fig A below; Fig. 9F in our revised manuscript). Previous studies have shown that mutations in conserved amino acid residues within MYB domains can lead to significant alterations in their functional properties, underscoring the importance of these conserved sites (Guehmann et al., 1992, *Nucleic Acids Res* 20: 2279-2286; Yang et al., 2021, *Plant Physiol Biochem* 162: 124-136; Wang et al., 2022, *Nat Plants* 8: 1108-1117).

The [DE]Lx2[RK]x3Lx6Lx3R sequence, located within the MYB domain, is a critical region for the interaction of R3-MYBs with bHLH proteins. Notably, D34 represents the first amino acid of this sequence (Simon et al., 2007, *Dev Biol* 311: 566-578; Matsui et al., 2008, *Plant J* 55: 954-967). Additionally, M68 has been shown to be essential for the function and intercellular movement of CPC proteins (corresponding residue M78 in CPC) (Kurata et al., 2005, *Development* 132: 5387-5398). Therefore, we hypothesize that the variations at these two loci may contribute to the functional specificity of PavMYB.C2 in sweet cherry, potentially modulating its role in anthocyanin regulation.

Subsequently, we generated two mutated versions of PavMYB.C2: PavMYB.C2_D34, in which the amino acid T34 was substituted with D34, and PavMYB.C2_M68, in which the amino acid S68 was substituted with M68 (See Fig B below; Fig. 9G in our revised manuscript). These mutated constructs were then introduced into the pGreenII 62SK vector as effector constructs. Both constructs were co-transfected into tobacco leaf protoplasts along with the proUFGT:LUC reporter plasmid, respectively. Our results indicated that co-transfection of the proUFGT:LUC reporter plasmid with the 35S:PavMYB.C2 or PavMYB.C2_D34 plasmid resulted in a significant increase in relative LUC activity compared to the control, However, when the 35S:PavMYB.C2_M68 plasmid was co-transfected, the transcriptional activation capacity was absent (See Fig C below; Fig. 9H in our revised manuscript). These findings suggest that the activation of *UFGT* by PavMYB.C2 is dependent on the S68 site, rather than the T34 site. Based on these data, we propose that the functional specificity of PavMYB.C2 in sweet cherry may be attributed to the variations at the S68 site, which play a critical role in its ability to regulate anthocyanin biosynthesis.

We have now integrated these findings into the results section and have made corresponding modifications to the methods. Additionally, the functional specificity of PavMYB.C2 is further discussed in the discussion section of the manuscript. We would also like to acknowledge that the reviewer have provided us with valuable insights, leading to a new avenue of research to explore the underlying mechanism of PavMYB.C2’s functional specificity in future studies. In particular, we recognize that the mutation from methionine (M) to serine (S) results in the substitution of non-polar hydrophobic amino acids by polar hydrophilic residues, this could potentially affect the spatial conformation of the protein. This alteration may affect its affinity for interactions with other proteins or DNA. In future work, we may consider exploring protein crystallization and structural determination techniques as previously reported (Wang et al., 2022, *Nature Plants* 8: 1108-1117), expanding beyond our current focus on molecular experiments.

**Regulation of the *UFGT* by PavMYB.C2 is dependent on the S68 site of PavMYB.C2.** (A) Multiple protein sequence alignment of PavMYB.C2 and related MYBs from *Arabidopsis*. The red boxes highlight amino acids that are conserved in *Arabidopsis* MYBs but differ in PavMYB.C2. D: Aspartic acid; T: Threonine; M: Methionine; S: Serine. (B) Diagrams showing the PavMYB.C2 variants. PavMYB.C2_D34, a mutated version of PavMYB.C2 with the amino acid T34 substituted by D34; PavMYB.C2_M68, a mutated version of PavMYB.C2 with the amino acid S68 substituted by M68. (C) Effects of PavMYB.C2, PavMYB.C2_D34, and PavMYB.C2_M68 on the activation of *UFGT*. The empty pGreenII 62SK vector served as the negative control.

**Answers to Reviewer #2**

Anthocyanins are vital secondary metabolites responsible for fruit coloration and health benefits. Thus, it is the key step to explore the genetic mechanisms regulating anthocyanin biosynthesis in fruits. It is the case reported in this manuscript. Using cherry fruit as materials, the authors identified a PavMYB.C2-UFGT module, and provide solid evidence to support this module contributing to fruit coloration via modulating anthocyanin biosynthesis.

**Answer:** We are grateful to you for your appreciation of the value of our work. We thank you very much for your detailed suggestions and comments.

Checking the legend of Fig 5, it is not right for B-G. In addition, it is better to express the unit of enzyme activity based on mg protein, instead of fresh weight.

**Answer:** Thanks for the reviewer's meticulous observation and correction of our inaccurate description. We have now carefully revised the legend of Fig 5 to accurately reflect the order of the images. Furthermore, we acknowledge the oversight in the labeling of enzyme activity units in Figs 5F-H. After careful review, we confirm that the data should be expressed as “U/g protein” rather than based on fresh weight (FW), and we have now made the appropriate correction.

It is recommended to verify the function of UFGT in cherry fruit as PavMYB.C2.

**Answer:** We thank the reviewer for the valuable suggestion. To further validate the role of UFGT in anthocyanin biosynthesis in cherry fruit, we have constructed fusion vectors for *UFGT* overexpression (35S:UFGT) and virus-induced gene silencing (pTRV2:UFGT). Agrobacterium strain GV3101 carrying these recombinant plasmids was introduced into fruit at 20 days post-anthesis (DPA) (See Fig A below; Fig. 9B in our revised manuscript). Successful genetic transformation was confirmed by RT-qPCR analysis 15 days post-injection (See Fig B below; Fig. 9C in our revised manuscript).

Phenotypic observations showed that *UFGT* overexpression (OE) fruits exhibited a deeper red coloration compared to controls, while *UFGT* silencing (VIGS) fruits appeared greener, similar to the phenotype induced by PavMYB.C2 (See Fig A below; Fig. 9B in our revised manuscript). Furthermore, *UFGT*-OE fruits showed a significant increase in Cy3R and Cy3G levels, with Cy3G accumulation surpassing that of Cy3R. In contrast, these anthocyanins were markedly reduced in *UFGT*-VIGS fruits (See Fig C and D below; Fig. 9D and E in our revised manuscript). These results confirm the essential role of UFGT in anthocyanin accumulation in sweet cherry fruit.

We have now incorporated these findings into the results section and modified the methods accordingly.

**Transient genetic transformation of the *UFGT* gene in sweet cherry fruit.** (A) Transient genetic transformation in sweet cherry fruit. 'OE' denotes transient overexpression of the *UFGT* gene, 'VIGS' refers to *UFGT* gene silencing, and 'Empty' indicates the injection of a corresponding empty vector. Images were captured 15 days post-injection. (B) Relative transcript levels of *UFGT* in 'Empty', 'OE', and 'VIGS' fruits 15 days after injection. (C and D) Quantification of two major anthocyanins in 'Empty', 'OE', and 'VIGS' fruits 15 days post-injection. D: Cyanidin-3-O-rutinoside (Cy3R); E: Cyanidin-3-O-glucoside (Cy3G).

In Fig 6C, it is better to include a nucleus marker or DAPI.

**Answer:** Following the reviewer’s suggestion, we have now performed a subcellular localisation assay using a nuclear localization marker (nuclear localization-mCherry). The results clearly demonstrate that PavMYB.C2 is predominantly localized to the nucleus (See Fig blow; Fig. 6C in our revised manuscript), which is consistent with our previous findings. We have updated the legend for Fig 6C and revised the corresponding methods section to reflect this new information in the revised manuscript.

**Subcellular localization of PavMYB.C2.** Recombinant vectors (PavMYB.C2-eGFP and free-eGFP) were introduced into *Nicotiana benthamiana* mesophyll cells. Subcellular localization was determined by observing GFP fluorescence under a fluorescence microscope, with co-expression of a nucleus-targeted mCherry marker to delineate nuclear regions. Scale bar = 20 μm.

**Answers to Reviewer #3**

The study employs a comprehensive multi-omics approach, integrating metabolomics, transcriptomics, and functional validation, to explore the regulatory mechanisms underlying anthocyanin biosynthesis in sweet cherry. The identification of PavMYB.C2 as a pivotal transcriptional regulator for UFGT and anthocyanin accumulation represents a novel advancement in this field. Functional validation through transient overexpression and silencing experiments convincingly highlights the role of PavMYB.C2 in modulating anthocyanin composition, particularly cyanidin-3-glucoside (Cy3G), thereby enhancing our understanding of MYB transcription factors in anthocyanin regulation. However, several issues need to be addressed before publication.

**Answer:** We are grateful to you for your appreciation of the value of our work. We thank you very much for your detailed suggestions and comments.

Major Comments

The authors demonstrate that PavMYB.C2 is a key transcriptional regulator of UFGT, which results in differing anthocyanin accumulation between the 'Binghu' and 'Isabella' cultivars. However, it is noted that 'Binghu' exhibits a less pronounced increase in the expression levels of DFR, ANS, and UFGT, whereas these genes are markedly upregulated in 'Isabella'. This indicates that apart from UFGT, the varying expression levels of DFR and ANS also contribute to the differential anthocyanin accumulation between the two cultivars. Please discuss potential contributors other than PavMYB.C2.

**Answer:** We thank the reviewer for the insightful comments. As noted, our study primarily focuses on PavMYB.C2 as a key transcriptional regulator of *UFGT*, which plays a crucial role in the observed differences in anthocyanin accumulation between the two cultivars. However, we acknowledge that the expression of other genes, particularly *DFR* and *ANS*, also appears to be a contributing factor to the variation in anthocyanin biosynthesis.

In this regard, we recognize that additional transcription factors in the blue module in addition to PavMYB.C2, such as PavWRK50, PavWRK47, PavNAC40, PavNAC14, PavERF113, and others (Fig. 5B and Data S5 in our revised manuscript), may influence the expression of *DFR* and *ANS*, thus affecting anthocyanin biosynthesis. Moreover, post-transcriptional modifications, including miRNA -mediated regulation, and environmental factors may also play important roles in the cultivar-specific differences observed.

We have now included a discussion of these potential contributors in the revised manuscript, providing a more comprehensive understanding of the regulatory network underlying anthocyanin accumulation. We believe this additional information enhances the manuscript.

Given that PavMYB.C2 regulates the synthesis of cyanidin-3-O-glucoside and overall anthocyanin production, what could explain its differential expression between the two cultivars despite their similar genetic backgrounds?

**Answer:** Thank you for your insightful comment. The differential expression of *PavMYB.C2* between the two cultivars, despite their similar genetic backgrounds, can be attributed to several potential factors.

**Epigenetic Regulation:**

Differences in DNA methylation, histone modifications, or chromatin accessibility at the PavMYB.C2 site could modulate its transcription. Epigenetic variations are well-documented drivers of gene expression divergence in closely related cultivars, even in the absence of genetic sequence differences (Tang et al., 2022, *New phytologist* 228: 839-844; Zhang et al., 2023, *Nature Communications* 14:8238).

**Post-Transcriptional Regulation:**

Differences in miRNA or RNA-binding protein activity could affect PavMYB.C2 mRNA stability or translation efficiency, leading to disparities in protein levels even with comparable transcript abundance (Ren et al., 2012, *PNAS* 109: 12817-12821).

**Transcriptional Cascade Regulation:**

Differential expression might arise from variations in the interaction with other transcription factors and co-factors. As suggested by our co-expression network analysis (Fig. 5B and Data S5 in our revised manuscript), transcription factors like PavWRK50, PavWRK47, PavNAC40, and PavNAC14 may modulate the expression of genes involved in anthocyanin biosynthesis, and differences in their activity between cultivars could affect *PavMYB.C2* expression.

Experimental Timing for Agrobacterium Infiltration:The manuscript describes agrobacterium infiltration at 20 days post-anthesis (DPA) and sample collection fifteen days post-infiltration. Why were these specific time points chosen?

**Answer:** Thank you for your question. Infiltration at 20 days post-anthesis (DPA) targets a pre-ripening stage when the fruit undergoes active cell expansion and metabolic preparation for ripening, ensuring optimal tissue receptivity to Agrobacterium. This timing precedes the natural fruit turns colour (~30–45 DPA), allowing the overexpression of *PavMYB.C2* to influence the pathway before its natural activation, thereby enhancing measurable effects.

Sampling at 15 days post-infiltration (35 DPA total) aligns with the early ripening stage, when anthocyanin accumulation typically begins to increase rapidly. This timing allows for effective detection of PavMYB.C2’s regulatory influence. The chosen interval provides sufficient time for gene expression and phenotypic changes while avoiding potential confounding factors, such as fruit senescence, which emerge at later ripening stages.

EMSA Data and Binding Motifs:The EMSA data (Fig 8D) show binding to MBS3 but not MBS1/MBS2. However, the rationale for selecting these motifs and their conservation across species is unclear. A phylogenetic analysis of the UFGT promoter region or comparison with known MYB-binding motifs in other plants (if available) would strengthen this claim.

**Answer:** We understand the reviewer’s concerns. In this study, the motifs MBS1, MBS2, and MBS3 were identified through promoter analysis using online tool PlantCARE (<http://bioinformatics.psb.ugent.be/webtools/plantcare/html/).> Notably, previous studies have demonstrated the differential affinity of MYB transcription factors for distinct *cis*-acting elements within the promoter regions of target genes (Phan et al., 2011, *Plant Cell* 23:2209-2224; Shang et al., 2022, *Life Science Alliance* 5:e202201450). This variation in binding affinity may be influenced by a range of factors, including the structural characteristics of the MYB protein, epigenetic modifications, as well as the presence of other transcription factors or cofactors that can influence the binding process. We hope this explanation addresses the reviewer’s concerns.

Data Availability:While the manuscript states that“all relevant data are within the manuscript and Supporting Information,” raw transcriptomic and metabolomic datasets should be deposited in public repositories (e.g., NCBI GEO) to ensure reproducibility.

**Answer:** The raw omics data reported in this paper have been deposited in the National Genomics Data Center (NGDC), China National Center for Bioinformation under the accession numbers CRA021570 and OMIX009876.

Some references related to anthocyanins are outdated and need to be updated with more recent literature

**Answer:** Thanks for the suggestion. We have carefully reviewed the relevant literature and updated the manuscript with more recent studies. The newly cited references are as follows:

Lu Z, Wang X, Lin X, Mostafa S, Zou H, Wang L, et al. Plant anthocyanins: Classification, biosynthesis, regulation, bioactivity, and health benefits. Plant Physiol Biochem. 2024;217: 109268.

Zhang D, Zhou H, Zhang Y, Zhao Y, Zhang Y, Feng X, et al. Diverse roles of MYB transcription factors in plants. J Integr Plant Biol. 2025; 67(3): 539-562.

Ma M, Li R, Li Y, Dai W, Shang J, He Y, et al. Anthocyanin biosynthesis and transport synergistically modulated by RcMYB75 and RcGSTFL11 play a pivotal role in the feedforward loop in response to drought stress. Plant J. 2025;121(3): e17240.

Du H, Zhai Z, Pu J, Liang J, Wang R, Zhang Z, Wang P, Zhu Y, Huang L, Li D, Chen K, Zhu G, Zhang C (2025) Two tandem R2R3 MYB transcription factor genes cooperatively regulate anthocyanin accumulation in potato tuber flesh. Plant Biotechnol J

Zhang Z, Wang X, Gao Y, Xian X, Zhang D, Zhao W, et al. Orchestrating anthocyanin biosynthesis in fruit of fruit trees: Transcriptional, post-transcriptional, and post-translational regulation. Int J Biol Macromol. 2025;307(Pt 1): 141835.
