# Supplementary material for: The PavMYB.C2-UFGT module contributes to fruit coloration via modulating anthocyanin biosynthesis in sweet cherry
Source: PLoS Genet. 2025 Jun 17;21(6):e1011761. doi: 10.1371/journal.pgen.1011761 (PMC12185008; doi:10.1371/journal.pgen.1011761)
Supplement: Attachment — Submitted filename: Response_to_Reviewers_auresp_2.doc [file pgen.1011761.s015.doc]

**Answers to Editors**

Reviewer 1 has raised two important points. While the authors have provided some explanations, these could be clarified and emphasized further. Please consider the following suggestions as minor revisions:

1. Based on the presented data (phylogenetic tree as well as Myb domain AA conservation), PavMYB.C2 appears to have diverged significantly from both the AtCPC and PavMYB.C1 supgroup. Moreover, unlike AtCPC, which functions as a repressor, PavMYB.C2 acts as an activator. These functional and evolutionary differences should be more clearly highlighted and emphasized in the manuscript to avoid confusion. This clarification also addresses Reviewer 1’s second concern.

**Answer:** We appreciate the editor's insightful suggestion. We have revised the text to explicitly highlight PavMYB.C2’s evolutionary divergence from AtCPC (see revised Results section) and its functional role as an activator, contrasting it with AtCPC’s repressor activity (see the Discussion section and other minor changes). These changes clarify the uniqueness of PavMYB.C2 and address potential confusion.

2. Additionally, please clarify whether Figure 6B is based on the full-length amino acid sequence or only the MYB domain. Have you also analyzed sequence homology at the DNA level? More detailed data and a clearer description of how homology was assessed (AA vs. DNA vs. domain-based) would strengthen the manuscript and aid reader understanding.

**Answer:** We sincerely thank the editor for the insightful and valuable suggestion. We have clarified that Figure 6B is based on full-length amino acid sequences (see revised Figure 6B legend) and added methodology details for homology assessment (see revised Methods). Following the editor's suggestion, we have also constructed phylogenetic trees using the MYB domain amino acid sequence and the full-length coding DNA sequence (CDS) to assess sequence homology. The results showed that the clustering patterns of the phylogenetic trees were consistent with those obtained from the full-length amino acid sequences, wherein PavMYB.C2 exhibited distinct differences from AtCPC, thereby further highlighting the specificity of PavMYB.C2. We have incorporated a corresponding description of these findings in the Results and Methods sections, and the additional figures are now included as Supplemental Figure S3 in the revised manuscript.

**S3 Fig. Phylogenetic analysis of the LOC110764247 (PavMYB.C2) homologs in *Arabidopsis thaliana* and *Prunus avium*.**

(A) Phylogenetic tree constructed using amino acid sequences of MYB domains.

(B) Phylogenetic tree constructed using full-length coding DNA sequences.

**Answers to Reviewer #1**

The authors performed a number of experiments to demonstrate that PavMYB.C2 positively regulates the expression of UFGT in sweet cherry, thereby influencing color development. However, I still have some concerns and questions:

It is unclear why the authors chose to test the effect of Arabidopsis transcription factors, such as CPC and ETC, on sweet cherry UFGT. Did they not identify any orthologs of these factors in sweet cherry? If suitable orthologs were absent, the relevance of testing Arabidopsis transcription factors in this context is questionable and may not yield biologically meaningful insights.

**Answer:** Thank you for your thoughtful comments. As the editor rightly pointed out above, Figure 6B and the results of our newly added analyses confirm that in sweet cherry, PavMYB.C1 is homologous to the CPC proteins, while PavMYB.C2 exhibits distinct differences from both AtCPC and PavMYB.C1 subgroup. This distinction further underscores the unique specificity of PavMYB.C2. The functional differences between MYB.C2 and CPC proteins have been thoroughly documented in both the manuscript data and in the previous version of our response letter.

I am also curious whether the authors investigated the potential role of PavMYB.C2 in forming or inhibiting the MBW complex, which is well-known to regulate anthocyanin biosynthesis in sweet cherry. I strongly recommend performing such experiments to better understand the mechanistic function of PavMYB.C2 within the regulatory network.

**Answer:** We thank the reviewer for the comments. Our study have emphasized the independent regulatory role of PavMYB.C2 in modulating anthocyanin levels in sweet cherry fruits. While understanding its interaction with the MBW complex is important for a comprehensive view of its regulatory function, such experiments would require extensive experiments, including protein interaction assays, MYB/bHLH/WD40 complex identification, genetic transformation, and perennial fruit tree sampling, among other considerations. Given the complexity, these investigations are better suited for future studies. In the meantime, we have highlighted the significance of the MBW complex in the Discussion section and are actively pursuing further research on the regulatory network involving PavMYB.C2.
